# Ionotropic GABA Receptors and Distal Retinal ON and OFF Responses

**DOI:** 10.1155/2014/149187

**Published:** 2014-07-20

**Authors:** E. Popova

**Affiliations:** Department of Physiology, Medical Faculty, Medical University, 1431 Sofia, Bulgaria

## Abstract

In the vertebrate retina, visual signals are segregated into parallel ON and OFF pathways, which provide information for light increments and decrements. The segregation is first evident at the level of the ON and OFF bipolar cells in distal retina. The activity of large populations of ON and OFF bipolar cells is reflected in the b- and d-waves of the diffuse electroretinogram (ERG). The role of gamma-aminobutyric acid (GABA), acting through ionotropic GABA receptors in shaping the ON and OFF responses in distal retina, is a matter of debate. This review summarized current knowledge about the types of the GABAergic neurons and ionotropic GABA receptors in the retina as well as the effects of GABA and specific GABA_A_ and GABA_C_ receptor antagonists on the activity of the ON and OFF bipolar cells in both nonmammalian and mammalian retina. Special emphasis is put on the effects on b- and d-waves of the ERG as a useful tool for assessment of the overall function of distal retinal ON and OFF channels. The role of GABAergic system in establishing the ON-OFF asymmetry concerning the time course and absolute and relative sensitivity of the ERG responses under different conditions of light adaptation in amphibian retina is also discussed.

## 1. Introduction

In the vertebrate retina, visual information is processed into parallel ON and OFF pathways, which carry information for light increments and decrements, respectively (for review, [1–3]). The existence of two parallel output pathways has advantages in allowing small signals to remain prominent over a greater dynamic range. The ON-OFF segregation begins with the divergence of photoreceptor signals to two subclasses of bipolar cells: ON and OFF types [4]. The activity of the ON and OFF bipolar cells is reflected in the b- and d-waves of the diffuse electroretinogram (ERG) obtained with long lasting light stimuli. The electroretinogram provides an excellent noninvasive tool to assess function of the distal retinal ON and OFF channels in humans and animals. Studies performed mainly on nonmammalian retina have revealed some asymmetries in the ERG ON and OFF responses, but their origin is not well understood [5–11]. One potential source of these asymmetries is a different role, played by the retinal inhibitory neurotransmitter systems in their generation. GABA is the major inhibitory neurotransmitter in the vertebrate retina. Its physiologic actions are mediated by three types of membrane receptors: ionotropic GABA_A_ and GABA_C_ receptors and metabotropic GABA_B_ receptors. This review concentrates on the effects of GABA and specific antagonists of the ionotropic (GABA_A_ and GABA_C_) receptors on the ERG b- and d-waves in both mammalian and nonmammalian retina. The author has included her own results demonstrating that some of the asymmetries in the ERG ON and OFF responses, obtained under different conditions of light adaptation, are indeed due to the GABAergic system. In the review are also presented many data concerning the types of the GABAergic neurons and ionotropic GABA receptors in the vertebrate retina and their role in shaping the light responses of the ON and OFF bipolar cells.

## 2. GABAergic Neurons in Retina

Gamma-aminobutyric acid (GABA) fulfills all of the criteria needed to establish a substance as a neurotransmitter in the retina. GABA is present in high concentration in some retinal neurons, which have high activity of L-glutamate decarboxylase (GAD, the major synthesizing enzyme for GABA) and high-affinity uptake system for GABA to terminate its transmitter action. They release GABA during depolarization or in response to a number of stimuli including light. GABA receptors have been well demonstrated in the retina (reviews: [12–15]).

### 2.1. GABAergic Amacrine Cells

**Figure 1:**
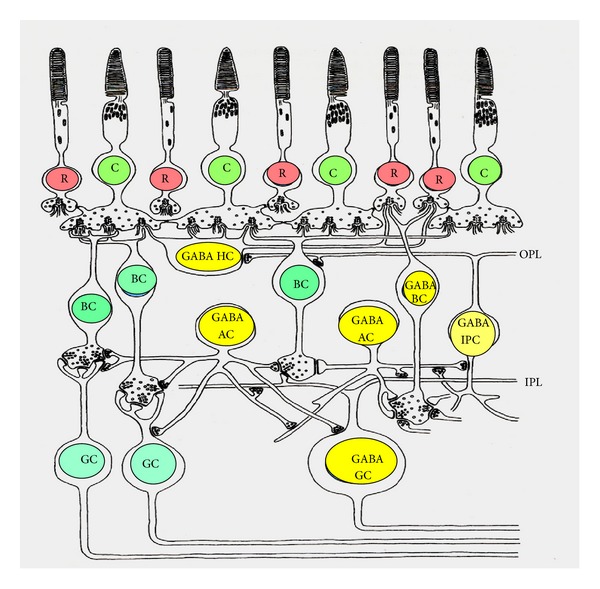
Diagram of the synaptic organization of the retina showing all types of GABAergic neurons. R, rod; C, cone, BC, bipolar cell; HC, horizontal cell; AC, amacrine cell; GC, ganglion cell; GABA HC, GABAergic horizontal cell; GABA AC, GABAergic amacrine cell; GABA IPC, GABAergic interplexiform cell; GABA GC, GABAergic ganglion cell; OPL, outer plexiform layer; IPL, inner plexiform layer.

All vertebrate species have a large population of GABAergic retinal neurons identified as amacrine cells and displaced amacrine cells (Figure 1). The GABAergic amacrine cells form a dense and heterogeneous population of cells branching at all levels of the inner plexiform layer (IPL). They are of ON, OFF, or ON-OFF functional types. GABAergic amacrine cells receive their synaptic input predominantly from bipolar cells, while their second most common input is from other amacrine cells including GABAergic ones [16, 17]. The GABAergic amacrine cells make conventional synapses onto bipolar and amacrine cell processes, as well as onto the somata and dendrites of ganglion cells (*fish*: [18];* rabbit*: [19–21]). The synaptic output is greatest to cone and rod bipolar axon terminals (*fish*: [18];* rat*: [16];* cat*: [22];* monkey*: [23, 24]), where GABAergic amacrines provide both reciprocal (direct) and lateral (nonreciprocal) feedback to bipolars. It has been shown that there are relatively more GABAergic amacrine to bipolar synapses in the ON than OFF sublaminae of the IPL in monkey retina [24]. The same is true for the GABAergic amacrine to ganglion cells synapses in monkey [24] and cat retina [17, 25]. Interesting ON-OFF asymmetry has been reported in* Bufo marinus* retina, where synaptic contacts of GABAergic amacrines with bipolar cells are more frequent in the OFF-sublamina, and those with ganglion cell dendrites in the ON-sublamina [26]. GABA is released from amacrines by depolarizing stimuli in a calcium-dependent manner (review: [12]). It has been shown that this release is inherently slow and it becomes more transient after increasing slow Ca^2+^ buffering or blocking L-type Ca^2+^ channels and Ca^2+^ release from intracellular stores [27]. Eggers et al. [27] suggest that the GABAergic amacrine cells have a prolonged buildup of Ca^2+^ in their terminals that causes slow, asynchronous release. This could be a mechanism of matching the time course of amacrine cell inhibition to bipolar cell glutamate release. In addition to this conventional calcium-dependent mechanism, GABA may be released from amacrine cells in a calcium-independent manner. It has been shown that cells containing GAD-67 release GABA via its transporter, while cells containing exclusively GAD-65 apparently do not release the neurotransmitter via the transporter [28].

The GABAergic amacrine cells comprise many different morphological classes in both mammalian ([29, 30]; review: [31]) and nonmammalian retina [32–36]. They are all of medium- to large-field types and are presumed to mediate lateral inhibition in the inner plexiform layer, typically within a single layer (review: [37]). The latter process has been implicated in the formation of antagonistic surrounds of certain ganglion cells [38, 39] and complex response properties of ganglion cells, such as direction or orientation sensitivity [40–43]. Most of the data indicate that GABAergic amacrine cells are not involved in the crossover inhibition between ON and OFF channel, although some exception from this rule may exist [44, 45].

### 2.2. GABAergic Interplexiform Cells

In mammalian retina some of the GABAergic neurons in the inner nuclear layer are GABAergic interplexiform cells (*rodent*: [32, 46–49];* cat*: [17, 25, 32, 46, 50];* rabbit*: [32, 51];* primate*: [28, 52, 53];* human*: [32, 54, 55]) (Figure 1). Although most of them contain a second neurotransmitter (dopamine, acetylcholine, NO) as well as GABA, types of interplexiform cell containing only GABA have also been described [49, 53]. Some of these cells stratify in stratum S4/5 of the inner plexiform layer and send processes to the outer plexiform layer [49]. They are of ON functional type and their responses to green (578 nm) and blue (400 nm) light suggest that they receive input from cone bipolar cells contacting both M- and S-cones. GABAergic interplexiform cells receive most of their synaptic inputs from amacrine cells in IPL and provide synaptic outputs to both IPL and OPL (review: [12]). Output in the outer plexiform layer is mostly onto rod bipolar cells, with an occasional cone bipolar cell [56]. In the inner plexiform layer they synapse on amacrine cells and both rod and cone bipolar cells [57]. The functional role of the GABAergic interplexiform cells has not yet been established.

### 2.3. GABAergic Horizontal Cells

There is strong evidence that GABA is neurotransmitter of some types of horizontal cells in nonmammalian retina (reviews: [12, 13, 36, 58]). Nonmammals have between two and five types of horizontal cells and only one type of cone-driven horizontal cell (H_1_) is GABAergic in fish and turtle retina ([59–61]; review: [62]). In amphibian retina H_1_ GABAergic cells receive mixed rod-cone input [63]. GABAergic horizontal cells have also been described in some mammalian species (*rodent*: [47, 64, 65];* cat*: [17, 25, 32];* rabbit*: [47, 51, 66];* monkey*: [29, 67, 68]) (Figure 1). Most mammals have two types of horizontal cells, and both are GABAergic (review: [62]). It is still unclear how the GABAergic horizontal cells release GABA. In nonmammalian retina most of the GABA efflux from horizontal cells is Na^+^-dependent, Ca^2+^-independent, and controlled by voltage over a broad range. Only a small part of GABA efflux is Ca^2+^-dependent release (review: [62]). Thus, it appears that the major mechanism for GABA release is through Na^+^-coupled GABA transporters, which cause release of GABA during darkness, when the horizontal cells are depolarized, but accumulation of GABA in the cytoplasm during continuous illumination, when the horizontal cells are hyperpolarized. Mammalian horizontal cells do not appear to possess plasmalemmal Na^+^-dependent GABA transporters and the cellular mechanisms underlying transmitter release from these cells are not fully understood. It is thought that they release GABA in conventional vesicular manner [64, 69, 70]. The presence of SNARE proteins (syntaxin-4, syntaxin-1a, and SNAP-25), vesicular GABA transporters, and other presynaptic proteins in horizontal cell dendrites supports this idea [71–75]. It is proposed that mammalian horizontal cells release GABA during long periods of darkness, when they are relatively depolarized [62, 76]. Thus, it appears that both nonmammalian and mammalian horizontal cells release GABA in the dark, although by different release mechanisms.

The functional role of the GABAergic horizontal cells is not yet resolved. A hypothesis exists that the GABAergic horizontal cells mediate negative feedback to cones and thus are involved in surround mechanism generation in the outer plexiform layer. According to this hypothesis when the horizontal cells are depolarized in the dark, they release GABA, which opens Cl^−^ channels in cones. This leads to hyperpolarization and decrease release of neurotransmitter from the cones. In light the GABA release diminishes and thus it causes depolarization and increased release of photoreceptor neurotransmitter, which counteracts the effect of light on the cones [77, 78]. This hypothesis is supported by the observed voltage-dependent GABA release from horizontal cells [79] and the existence of GABA gating chloride channels in cones [80–82]. However, many data do not support this hypothesis. It has been shown that the surround illumination increases the chloride conductance in cones [83–86] instead of decreasing it [87, 88]. Moreover, the depolarizing responses of cones evoked by surround illumination, which are due to antagonistic synaptic inputs from horizontal cells, are not blocked or only partially blocked by GABA agonists and antagonists [85, 86, 88–91]. The latter observations call into question the involvement of GABA in negative feedback to cones. It has been shown that the horizontal cell feedback operates via a nonvesicular mechanism that shifts the voltage activation curve of calcium channels on cone axon terminals to more negative potentials [85, 92–94]. This shift opens calcium channels, causing a depolarization that opposes the light-induced photoreceptor hyperpolarization. Two mechanisms have been proposed to account for such a shift in voltage activation curve of calcium channels: (1) emphatic modulation of the local membrane potential in the cone terminal and (2) alteration in protons concentration in the synaptic cleft (for review: [95]). None of them is generally accepted. Another possible role of the GABAergic horizontal cells is their involvement in direct feed-forward synaptic inputs to bipolar cells which could account for antagonistic surround responses from bipolar cell receptive fields. This role has not yet been proved electrophysiologically (for review: [95]).

### 2.4. GABAergic Bipolar Cells

GABAergic bipolar cells have been found in amphibian [32, 33, 96, 97] and some mammalian species (*mouse*: [30];* cat*: [25, 50, 98, 99];* primate*: [67, 68, 76, 100];* human*: [67]) (Figure 1). The GABA containing bipolar cells are also glutamatergic and are found among both ON and OFF types in amphibian retina [101]. In mouse and cat retina they belong to OFF type bipolar cells [30, 99], while in monkey retina they are rod ON type bipolar cells [76, 100]. These differences suggest that the function of the GABAergic bipolar cells is species specific. Lassová et al. [76] proposed that the bipolar cells release GABA tonically, and the particular cell type that engages in this release depends on time of activity of the species: in monkey, a diurnal species, the release is mediated by ON bipolar cells; in cat, a nocturnal species, by OFF bipolar cells; and in salamander, a crepuscular species, by ON and OFF bipolar cells. In tiger salamander retina the GABAergic bipolar cells make more direct contacts with ganglion cells than the non-GABAergic (glutamatergic) bipolar cells [97]. These contacts are more frequent with OFF in comparison with ON ganglion cells. The GABAergic bipolar cells could be an origin of sustained inhibition involved in the push-pull modulation of ganglion cells responses observed in amphibians [102]. It has been shown that activation of bipolar cells by puffing KCl at their dendrites in OPL elicits inhibitory postsynaptic currents in some amacrine and ganglion cells. These inhibitory currents are picrotoxin sensitive, but resistant to blockers (CNQX and AP-5), which eliminate the responses of all third-order retinal neurons. Thus, they probably originate from GABA released by the GABAergic bipolar cells.

### 2.5. GABAergic Ganglion Cells

Although ganglion cells are glutamatergic in all vertebrate species, a small population of GABAergic ganglion cells have been found in both mammalian (*rat*: [32];* rabbit*: [32, 103];* cat*: [32];* monkey*: [104, 105];* human*: [32, 54, 55, 105]) and nonmammalian retina (*mudpuppy*: [32];* tiger salamander*: [33];* frog*: [26, 32];* turtle*: [32];* fish*: [32, 106];* chick*: [32, 107, 108]) (Figure 1). Some of the GABAergic ganglion cells colocalize glutamate and GABA [107]. GABA positive fibres have been seen in the optic nerve fiber layer [32, 33, 54], indicating that GABA may serve as an inhibitory transmitter of some retinal ganglion cells. It has been shown that GABA-immunoreactive amacrine cells synapse directly with GABA-immunoreactive ganglion cells [26] and thus may inhibit the centrally projecting inhibitory ganglion cells, causing disinhibition in the visual centres. The role played by the GABAergic ganglion cells in visual information processing is largely unknown.

## 3. GABA Receptors in the Retina

Physiological actions of GABA are mediated by three types of membrane receptors: ionotropic GABA_A_ and GABA_C_ receptors and metabotropic GABA_B_ receptors. The physiology of retinal ionotropic GABA receptors only will be discussed in the review.

### 3.1. GABA_A_ Receptors

**Figure 2:**
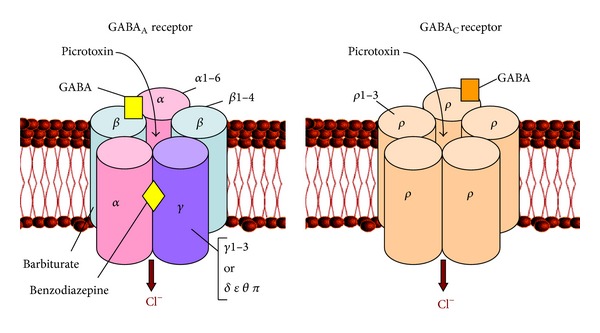
A schematic representation of ionotropic GABA receptors. Five subunits form a transmembrane chloride ion channel. The subunit composition of GABA_A_ (left) and GABA_C_ receptor (right) is indicated. The locations of the binding sites for GABA, GABA modulators (barbiturates and benzodiazepines) and antagonists (picrotoxin) are also shown.

GABA_A_ receptors are transmembrane proteins that consist of five subunits arranged around a central chloride ion-selective pore (review: [134]). They are heteromeric combination from seven main receptor subunit families: *α*, *β*, *γ*, *δ*, *ε*, *π*, and *θ* (review: [135]). Many of these subunits exist as multiple isoforms. To date six *α* (*α*1–6), four *β* (*β*1–4), three *γ* (*γ*1–3), and one *δ* subunit have been cloned. Each GABA_A_ receptor has two binding sites for GABA, which are located at the interfaces between the alpha and beta subunits (Figure 2). Selective agonists as muscimol, isoguvacine, THIP, and ZAPA bind to these binding sites. Competitive GABA_A_ receptor antagonists are bicuculline and SR 95531, while picrotoxin and TBPS are blockers of chloride channel [136, 137]. The GABA_A_ receptors have modulatory binding site for benzodiazepines, barbiturates, ethanol, and neurosteroids (review: [134]). Benzodiazepines bind to a pocket formed by the *α* and the *γ* subunits that is distinct from the agonist binding site located between one *α* and one *β* subunit [138, 139]. Thus, GABA_A_ receptors isoforms (*α* *β* *δ* and *α* *β* *ε*), which do not possess *γ* subunits, are benzodiazepine insensitive [140]. It has been shown that GABA_A_ receptors containing *α*_4_ and *α*_6_ subunit isoforms are also insensitive to benzodiazepines, because they contain an arginine residue instead of histidine at crucial position for benzodiazepine binding [141, 142]. When bound to their binding sites, benzodiazepines induce positive allosteric modulation of GABA_A_ receptors, which lead to increased sensitivity to GABA and increased frequency of chloride channels opening [136, 143, 144].

GABA_A_ receptors have been found in retina, where they mediate both presynaptic and postsynaptic inhibition (review: [145]). GABA_A_ receptors respond quickly to GABA, rapidly turning ON and OFF in several milliseconds (review: [37]). GABA_A_ receptor subunits have been localized on almost all retinal cells (Table 1). They are preferentially expressed in the inner plexiform layer, where they are clustered at postsynaptic sites of neurons (bipolar cell axons, amacrine cell processes, and ganglion cell dendrites), but they are also present in the OPL, localized on photoreceptors, horizontal cells, and bipolar cell dendrites. It has been shown that there are at least two functional subtypes of GABA_A_ receptor in the tiger salamander retina: one type is present only in the inner retina, primarily in the IPL, and is functionally coupled to benzodiazepine receptors; the other type is located in the OPL and is not coupled to the benzodiazepine receptors [146].

### 3.2. GABA_C_ Receptors

GABA_C_ or GABA_A_-rho receptors are ligand gated chloride channels, composed entirely of rho (*ρ*) subunits. To date, three *ρ* subunits have been identified - *ρ*_1_, *ρ*_2_, and *ρ*_3_, which form homopentameric (*ρ*1_5_, *ρ*2_5_, *ρ*3_5_) or heteropentameric complexes (*ρ*1_*m*_ *ρ*2_*n*_, *ρ*2_*m*_ *ρ*3_*n*_ where *m* + *n* = 5) [147, 148]. GABA_C_ receptors are selectively activated by (+)-cis-2-aminomethylcyclopropane-carboxylic acid [(+)-CAMP] and blocked by (1,2,5,6-tetrahydropyridin-4-yl)methylphosphinic acid (TPMPA). They are also blocked by picrotoxin, but not bicuculline. GABA_C_ receptors are not affected by many GABA_A_ receptor modulators such as barbiturates, benzodiazepines, and neuroactive steroids. It is known that GABA_C_ receptors are about 10-fold more sensitive to GABA than GABA_A_ receptors and thus are activated by lower concentrations of GABA [137, 149]. They respond more slowly to GABA and mediate more prolonged inhibitory signals [149].

The distribution of GABA_C_ receptors in the retina is more limited than GABA_A_ receptors. They are most abundant on bipolar cell axon terminals, but occasionally they have also been localized on cones, some horizontal cells, amacrine cells, and ganglion cells (Table 1). GABA_C_ receptors have been found also on bipolar cell dendrites, but their number is smaller than that obtained on axon terminals (*frog and turtle*: [111];* rat and rabbit*: [120];* monkey*: [120, 127]). The GABA_C_ receptors on bipolar cell axon terminals mediate large tonic inhibitory currents (reviews: [150–153]). The prolonged time course of this GABA_C_ feedback inhibition is particularly suited to regulate sustained exocytosis from bipolar cells [154] and thus to shape the temporal properties of the glutamate signal from bipolar cells to ganglion cells. GABA_C_ receptors are often found with GABA_A_ receptors on bipolar cell terminals, but they are not colocalized at the same synaptic sites [111, 121, 155], suggesting that they may be activated by distinct GABAergic cell types [152].

It has been shown that unique combinations of GABA_A_ and GABA_C_ receptors mediate inhibition in different classes of mammalian bipolar cells (review: [37]). In rod BCs, GABA-evoked currents are mediated primarily by GABA_C_ receptors. In cone ON bipolar cells, GABA_C_ receptors mediate most of the response, but there is a larger proportion of GABA_A_ receptor component compared to rod bipolar cells. In cone OFF BCs, there is about equal contributions of GABA_A_ and GABA_C_ receptors or the GABA_A_ receptor component is even greater than the GABA_C_ receptor component [153, 156–162]. It has been proposed that the temporal characteristics of inhibition, mediated by different proportions of GABA receptors, may be matched to the temporal characteristics of excitation in rod and cone bipolar cells [150]. A few studies have been performed to reveal the relative contribution of each receptor type to the overall GABA current elicited in the ON and OFF bipolar cells in nonmammalian retina. It has been shown that there is more GABA_A_-mediated currents than GABA_C_-mediated currents in axon terminals of ON bipolar cells, while the reverse is true for the OFF bipolar cells in frog retina [163]. Lukasiewicz et al. [164] argue that there may be a continuum in the complement of GABA receptors on bipolar cell axon terminals in tiger salamander retina. Most bipolar cells (8 of 13) seem to have a predominance of GABA_C_ receptors, some of them (3 of 13) seem to have a mix of GABA_C_ and GABA_A_ receptors, and few of them (2 of 13) seem to have a predominance of GABA_A_ receptors. However, they do not differentiate between the ON and OFF bipolar cells. Qian and Dowling [165] also have observed that the proportion of the GABA_C_ to GABA_A_ responses varies widely between bipolar cells in fish retina. More comparative studies of the GABA_A_ and GABA_C_ receptor elicited that currents in the ON and OFF bipolar cells are needed to elucidate their relative contribution to the overall GABA action on bipolar cells in nonmammalian retina.

## 4. Effects of GABA on Bipolar Cells

### 4.1. Effects on Bipolar Cell Axon Terminals

Bipolar cells in all vertebrates are very sensitive to exogenous GABA. GABA responses are more robust at axon terminals than dendrites and soma of the neurons, indicating that they receive the greatest GABAergic inhibitory input at this region [164, 166–171]. Axon terminals of ON and OFF bipolar cells are similarly sensitive to GABA [171]. GABA acting on ionotropic GABA receptors at axon terminals inhibits the calcium entry in the terminals and thus decreases the glutamate release from them [169, 172, 173]. It has been shown that the blockade of GABA_A_ and GABA_C_ receptors by picrotoxin increases the glutamate release from bipolar cells [174, 175] and augments the light-evoked excitatory postsynaptic currents in third-order retinal neurons [176]. It is not resolved yet if GABA inhibits the neurotransmitter release from both the ON and OFF bipolar cells. It has been found that the cone mediated light-evoked excitatory postsynaptic potentials are larger in ON, but not in OFF ganglion cells in transgenic mice lacking GABA_C_ receptors [161]. This indicates that GABA_C_ receptors inhibit the output from cone ON, but not from cone OFF bipolar cells in mouse retina.

Bipolar cells receive two types of GABAergic feedback inhibition at their axon terminal: reciprocal and lateral inhibition. Reciprocal inhibition is due to synaptic input from amacrine cells activated directly by the same bipolar cell, while lateral inhibition is due to synaptic input from amacrine cells activated by other bipolar cells. It has been shown that each mixed rod-cone ON bipolar cell terminal in goldfish retina receives ~350 inhibitory synapses from amacrine cells; ~50% are reciprocal and ~50% are lateral [184, 185]. The two types of inhibition may have distinct functional roles. Reciprocal inhibition is thought to make the output of BCs more transient [181], tuning it to the dynamic range of ganglion cells [154], preventing the rapid depletion of presynaptic vesicle pools [186], and modulating the excitatory output of bipolar cells onto ganglion cells' receptive field center [162, 187]. Lateral inhibition allows for spatial integration of signals [38], mediates center-surround organization of the receptive fields [188–190], and contributes to ganglion cell orientation selectivity [43]. The two types of inhibition are mediated by both GABA_A_ and GABA_C_ receptors. The GABA_A_ receptors mediate the phasic component, while GABA_C_ receptors mediate the tonic component of the response to GABA [150, 154, 157, 166, 175, 191–194]. It is true for both rod and cone bipolar cells [170, 195–198]. Serial inhibitory signals between homotype (ON-ON or OFF-OFF) and heterotype (ON-OFF or OFF-ON) GABAergic amacrine cells that are mediated by GABA_A_ receptors [44, 185] also affect the lateral inhibition on bipolar cell axon terminals [37]. It has been proposed that narrow-field light stimuli preferentially activate direct inhibition, while wide-field light stimuli preferentially activate serial inhibition [37]. Some data indicate that serial inhibition mediated by GABA_A_ receptors may limit the direct inhibition mediated by GABA_C_ receptors [37]. It has been shown that blocking of the GABA_A_ receptors with bicuculline increases the GABA_C_-mediated currents in bipolar cells [145].

### 4.2. Effects on Bipolar Cell Dendrites

Tonic inhibitory GABAergic currents have been well documented in bipolar cell dendrites in many mammalian and nonmammalian species [13, 116, 119, 127, 153, 158, 163, 183, 199]. It is thought that this inhibition generates the antagonistic responses from the receptive field surround, which are hyperpolarizing in ON bipolar cells and depolarizing in OFF bipolar cells. Because the dendrites of ON and OFF cone bipolar cells express the same type of GABA receptors [116, 117, 119, 158], the opposite polarity surround light responses may be generated by different chloride transporters expressed on bipolar cell dendrites. These chloride transporters regulate intracellular chloride levels and thus determine the chloride equilibrium potential (E_Cl_) in both types of bipolar cells. Some data indicate that ON bipolar cell dendrites express chloride accumulating Na–K–2Cl (NKCC) cotransporter and their E_Cl_ is more positive than the resting membrane potential. The OFF bipolar cell dendrites express chloride extruding K–Cl (KCC) cotransporter (it is expressed also on axon terminals of both bipolar types) and their E_Cl_ is more negative than the resting potential [200, 201]. Then, GABA released by horizontal cells in the dark could depolarize the dendrites of ON bipolar cells and hyperpolarize the dendrites of OFF bipolar cells by acting on Cl^−^ ionotropic channel. Illumination of the retina will diminish the release of GABA and opposite changes will result, hyperpolarization of ON bipolar cell dendrites and depolarization of OFF bipolar cell dendrites. However, there are no direct electrophysiological evidences supporting this hypothesis. Varela et al. [202] have shown that activation of GABA_A_ receptors located at the cell dendrites depolarized the rod ON bipolar cells, while activation of GABA_C_ receptors located at the cell axon terminal hyperpolarized the cells. The authors suggested that the physiological role of the GABA induced depolarization of rod bipolar cells is to enhance the contrast detection during scotopic vision, although rod bipolar cells do not show center-surround antagonism [203, 204].

### 4.3. Effects on Bipolar Cell Light Responses

Although all of the above presented data indicate that the retinal bipolar cells are targets of strong GABAergic influences in both their axon terminals and dendrites, the exact impact of these influences on their light responses is not yet well understood. In nonmammalian retina some authors have found that GABA diminishes the light responses of ON bipolar cells without changing their resting membrane potential (*mudpuppy*: [177, 205]). The effects of GABA persist after blockade of synaptic transmission with cobalt^+^ and are blocked by bicuculline and picrotoxin, indicating that they are due to direct action of GABA on ionotropic GABA receptors expressed on the ON bipolar cells. When antagonists of ionotropic GABA receptors are applied alone, they markedly enhance the amplitude of the light responses to both centre and surround illumination of the ON bipolar cells, which is associated with an increase of input resistance [177]. These findings support the idea that GABA antagonists block a continuous (dark) released GABA, which acts on the ON bipolar cells to increase their conductance and thus to shunt their light-evoked responses. This action of GABA probably does not take part in antagonistic centre-surround organization of their receptive fields, because the surround response is not eliminated by the GABA receptor antagonists (Table 2). Some authors argue that the effect of GABA on the amphibian ON bipolar cells may be related only to the time characteristics of the light responses and not to their amplitude. It has been shown that SR95531 (GABA_A_ antagonist) makes the responses slightly more transient without altering their amplitude, while picrotoxin makes them more sustained [206]. Most of the authors, who have investigated the effects of GABA and its antagonists on both the ON and OFF bipolar cells, reported for significant ON-OFF asymmetry (Table 2). Daniels [178] reported that bicuculline has no effect on the light responses of the mudpuppy OFF BCs, while it decreases the amplitude of the light responses of the ON bipolar cells and eliminates the antagonistic centre-surround organization of their receptive fields. Miller et al. [177] have demonstrated that although GABA by itself decreases the light responses of mudpuppy OFF BCs (similar to the ON BCs), GABA receptor antagonists bicuculline and picrotoxin do not alter the responses and have no effect on their centre-surround antagonism. The authors concluded that a GABA pathway is intimately related to the ON, but not to OFF bipolar cells in amphibian retina. An opposite ON-OFF asymmetry of the GABA action has been demonstrated in fish retina, where GABA produces a partial suppression of the light responses of the OFF BCs, while it has no effect on the ON BCs [180]. One may suggest that the inhibitory GABAergic synaptic inputs to the bipolar cells show species specific ON-OFF asymmetry. However, Stone and Schütte [179] have found that GABA causes a large reduction in the amplitude of the center responses of the OFF BCs and only a slight reduction of the center responses of the ON BCs in another amphibian species (*Xenopus*). The GABA effects on the surround responses show an opposite ON-OFF asymmetry, a minimal reduction in the OFF BCs responses and a substantial decrease in the ON BCs responses. Stone and Schütte [179] proposed that ON and OFF BCs may utilize different synaptic pathways for mediating centre-surround antagonism. The reduction of the OFF BC cell's center response may reflect direct synaptic input from GABAergic amacrine cells, while the reduction of the surround response of the ON BCs may be due to input from GABAergic horizontal cells. Other authors, who have studied the effects of GABA on the OFF BCs only, also have found that GABA reduces BC light responses in amphibian retina (*tiger salamander*: [182, 183]). Wu [183] has shown that GABA completely abolishes the surround response, while the center response was only decreased in amplitude. The actions of GABA, however, have been only observed in part of the preparations (45% in slices and 60% in the eyecups). These results are consistent with the idea that GABA may contribute in part to the surround responses of cone-driven OFF bipolar cells. It is important to note that bath application of agonists can activate nonsaturated GABA receptors throughout the retina, thereby complicating interpretation of the results. An opposite statement to that of Wu [183] has been made by Hare and Owen [182], who argued that GABA has no discernible effect on the receptive field organization of the OFF BCs in the same species. They have found that surround responses of OFF bipolar cells are not completely blocked by antagonists of GABA_A_, GABA_C_, and GABA_B_ receptors. Combinations of these drugs have been similarly ineffective in eliminating both the rod- and cone-driven surround responses [182]. The response amplitude of the centre and surround responses have been equally decreased by GABA receptor antagonists. GABA by itself also decreases the light responses of the OFF BCs. The ineffectiveness of the GABA antagonists to eliminate the surround responses of the OFF bipolar cells in these experiments may be due to inappropriate light adaptation conditions. The retina has been maintained under mid to high scotopic illumination conditions, while the bipolar cell surround responses are strongest under light adapted conditions [207, 208]. To reveal the role of the GABAergic system in the centre-surround organization of cone ON and OFF bipolar cells, the effects of GABA receptor antagonists should be determined under maintained light adapted conditions. Unfortunately, it has not yet been done.

A few studies have been performed to investigate the effects of GABA and its antagonists on the light responses of mammalian bipolar cells. It appears that the effect of GABAergic neurotransmission on the light-modulated currents of the rabbit bipolar cells depends on their type [209]. GABAergic antagonists have no effect on the OFF bipolar cells, but they suppress light-evoked cancelling inhibition in all rod bipolar cells. This canceling inhibition acts to suppress light-evoked excitation and thus to reduce the light response at all time scales. GABA antagonists suppress the light-evoked delayed cancelling inhibition, which occurs in about half of cone ON bipolar cells. The delayed inhibition truncates the light-evoked responses and makes them more transient [209]. Euler and Masland [181] have shown that the GABAergic inhibition on the rod ON bipolar cells in rat retina depends on the stimulus intensity. The rod ON bipolar cells respond with larger depolarization at lower but not higher stimulus intensities during the application of combination of bicuculline and TPMPA. This results in a steeper sensitivity curve and a decreased dynamic range of the responses. The authors concluded that the GABAergic system (via its action on axon terminals) is involved in widening the dynamic range of light responses in rod bipolar cells. It has been shown that the light-evoked inhibition upon the rod ON bipolar cell significantly decreases with light adaptation, which is due to decreased rod pathway activity as well as an increase in GABAergic inhibition between amacrine cells [210]. Together these serve to limit rod bipolar cell inhibition after light adaptation, when the rod pathway is inactive and modulation of the signal is not required. On the other hand, the dark adapted rod-dominant light responses of mouse OFF bipolar cells show a significant contribution of glycinergic inhibition, which decreases with light adaptation and is, surprisingly, compensated by an increase in the GABAergic inhibition [211]. The authors suggest that larger GABAergic input could reflect an adjustment of OFF bipolar cell spatial inhibition, which may be one mechanism that contributes to retinal spatial sensitivity in the light.

## 5. Effects of GABA on Diffuse Electroretinogram

Diffuse electroretinogram (ERG) consists of many components, but two of them are most prominent in response to long lasting stimuli: a b-wave (in response to stimulus onset) and a d-wave (in response to stimulus offset). These components are usually used for assessment of the retinal ON and OFF channel activity. The ERG b- and d-waves are thought to depend mainly on the activity of ON and OFF bipolar cells, respectively ([212–216]; reviews: [217, 218]). The role played by GABA in overall function of the distal retina can be easily investigated by application of exogenous GABA or blocking the retinal GABAergic neurotransmission and following up the changes of the ERG waves.

### 5.1. Effects of GABA on the Absolute Sensitivity of the ERG

The dependence of the absolute sensitivity of the ERG waves on the GABAergic neurotransmission in the retina still remains controversial. It has been shown that GABA inhibits or fully eliminates the scotopic threshold response, which appears below the b-wave threshold and is thought to originate in proximal mammalian retina (*rat*: [219];* mouse*: [220];* cat*: [221, 222]). The antagonists of ionotropic GABA receptors (picrotoxin and bicuculline) also reduce the scotopic threshold response in cat retina, thus acting in the same direction as GABA alone [221, 222]. However, the GABAergic blockade by bicuculline or 3-APA (GABA_C_ receptor antagonist) enhances the amplitude of the scotopic threshold response in rat retina [223]. Kapousta-Bruneau [223] concluded that both GABA_A_ and GABA_C_ receptors participate in the development of the scotopic threshold response and seemingly suppress it. They have proposed that a species difference could explain the contradiction between their results in rat and the results in cat [221, 222]. It appears that the role of the GABAergic neurotransmission in the scotopic threshold response generation is species specific in mammalian retina.

The influences of GABA and its antagonists upon the mammalian b-wave threshold have been investigated in a few studies. It has been shown that exogenous GABA lowers the scotopic b-wave threshold by 0.5 log units in cat retina, which means that GABA sensitizes the ERG ON response [221, 222]. Similar results have been obtained in mouse retina, where exogenous GABA causes a decrease of the b-wave amplitude, obtained with very low (threshold) intensities [228]. As it has been noted previously, the exogenously applied GABA can activate nonsaturated GABA receptors throughout the retina and thus complicate the interpretation of the results. In accordance with this statement are the results of Herrmann et al. [153], who have shown that although the exogenous GABA has no apparent effect on the absolute sensitivity of the rod-mediated b-wave, the sensitivity of the b-wave is decreased, when the GABA_C_ receptor function is eliminated (using GABA_C_R knockout mice or pharmacological blockade). Such effect has not been observed during the GABA_A_ receptor blockade. The authors concluded that endogenous GABA acts thought GABA_C_ receptors to sensitize the rod-mediated b-wave in mouse retina. It remains to be determined if GABA has such a role in the other mammalian species. There are no available data concerning the effects of the GABAergic system on the absolute sensitivity of the d-wave in mammalian retina.

In nonmammalian retina the action of endogenous GABA on the b-wave threshold appears to depend on the type of the photoreceptor input. The isolated GABA_A_ receptor blockade has no effect on the b-wave threshold, when the responses are mediated by red rods in mixed rod-cone amphibian retina (*mudpuppy*: [255];* frog*: [242]). Simultaneous GABA_A_ and GABA_C_ receptor blockade by picrotoxin also fails to alter the b-wave threshold, when the responses are mediated by red rods, but it decreases the b-wave threshold, when the responses are mediated by green rods or cones (*frog*: [242, 243, 260]). On the other hand, the d-wave threshold has been decreased by picrotoxin irrespective of the photoreceptor input. The effects on the d-wave threshold are stronger than those on the b-wave threshold, when the responses are mediated by cones, but they are of the same order, when the responses are mediated by green cones. These results indicate that the endogenous GABAergic system is essential for establishing the ON-OFF asymmetry in absolute sensitivity of the ERG responses, obtained under different conditions of adaptation and light stimulation. The same is true for the cone-dominated turtle retina. We have shown that the GABA_A_ receptor blockade increases the absolute sensitivity of b- and d-waves at all stimulus wavelengths, but the effect is strongest for blue ON and red OFF stimuli [250]. On the other hand, the GABA_C_ receptor blockade increases both the b- and d-wave sensitivity maximally to blue stimuli and to some lesser extent to green stimuli, while producing no change in the sensitivity to red stimuli [250]. The data obtained indicate that the absolute sensitivity of the ERG ON and OFF responses to different chromatic stimuli depends in a specific manner on the GABA_A_ and GABA_C_ receptor-mediated inhibition in turtle retina.

### 5.2. Effects of GABA and Benzodiazepines on Suprathreshold ERG

In mammalian retina some authors reported that exogenous GABA has no apparent effect on the scotopic and photopic b-wave amplitude (*rat*: [219]). The most of the other authors, however, have found that GABA increases the suprathreshold amplitude of the scotopic b-wave (*mouse*: [153, 228];* rabbit*: [229];* cat*: [221, 222]) (Table 3). The effect is evident over the entire dynamic range, and the semisaturation constant is shifted to the left by 0.5 log units, indicating that the relative sensitivity of the scotopic response is increased [221, 222]. These results indicate that GABA has sensitizing effect on the scotopic ON response. The opposite statement could be made from the results of other authors, who have shown that exogenously applied GABA decreases the amplitude of the scotopic b-wave (*rat*: [224];* rabbit*: [230, 231]). The suppressing effect of GABA is evident in the entire intensity range with a relative reduction of the b-wave amplitude being more prominent with higher light intensities [231]. Why exogenous GABA has diverse effects on the scotopic b-wave amplitude in one and the same species remains unclear. It seems unlikely that differences in the experimental procedure (in vivo or in vitro) could account for the reported inconsistency, since opposite results have been reported for in vitro rabbit preparations [229, 230].

Not many studies have been undertaken to reveal the effects of benzodiazepines on the mammalian ERG. No significant effects of benzodiazepines have been seen on the b-wave in human [261, 262], cat, and rat retina [263]. In contrast to the lacking effect on the ERG, Robbins and Ikeda [263] have demonstrated specific benzodiazepine binding sites restricted to the inner plexiform layer with minor amounts of binding in the inner nuclear and ganglion cell layers. The authors have concluded that “retinal benzodiazepine receptors do not influence visually induced preganglionic retinal activity.” Other authors, however, reported that diazepam decreases the amplitude of the b-wave in human [264, 265] and cat retina [266, 267]. The reduced b-wave amplitude in human ERG has been accompanied with significantly lengthened latency and implicit time of the response [264, 265]. Chynoransky [265] argue that the time characteristics of the response have been the most intensively affected. Lanoir et al. [266] have seen similar effects of benzodiazepines on cat ERG, while Schulze and Appel [267] have obtained that the decreased b-wave amplitude in cat ERG has been accompanied with unaltered implicit time. There are no available data concerning the effects of benzodiazepines on mammalian d-wave. Additional studies are needed to establish the role of benzodiazepine regulatory sites on retinal GABA_A_ receptors in shaping the mammalian ERG ON and OFF responses.

More consistent results have been reported for the effects of exogenously applied GABA on the* nonmammalian* ERG waves. It has been shown that GABA decreases the suprathreshold b-wave amplitude in chicken ERG [237]. The same effect has been observed for both the b-wave and d-wave amplitude in amphibian and reptile retina (*frog*: [111, 239, 240];* Xenopus*: [249];* turtle*: [111]). The effect does not depend on conditions of light adaptation, because it is evident in scotopic [239], mesopic [111, 240], and photopic [249] conditions. These results suggest that GABA has inhibitory action on both the ON and OFF ERG responses, which does not depend on the type of the photoreceptor input. How this inhibitory action is modulated by benzodiazepines, however, is largely unknown. We cannot find any study except our own, where the effects of benzodiazepines have been investigated on nonmammalian ERG. In our study we have shown that flurazepam decreases the amplitude of the b- and d-waves in frog ERG without changing their implicit time [240]. Flurazepam enhances the inhibitory effect of GABA on the amplitude of the ERG waves, which supports the suggestion for the existence of functional benzodiazepine regulatory sites on GABA_A_ receptors expressed on the ON and OFF bipolar cells in frog retina. We have demonstrated that the inhibitory effect of flurazepam on the b- but not d-wave amplitude is blocked by bicuculline, suggesting that benzodiazepine regulatory sites on the GABA_A_ receptors expressed on the ON bipolar cells are bicuculline-sensitive, while those on the OFF bipolar cells are bicuculline insensitive.

### 5.3. Effects of GABA Antagonists on Suprathreshold ERG

#### 5.3.1. Effects of GABA_A_ Receptor Antagonists

The role played by endogenous GABA acting on different types of ionotropic receptors can be easily evaluated by application of selective receptor antagonists and following up changes of the ERG waves. The application of GABA_A_ receptor antagonists (bicuculline, SR95103, SR95531) causes diverse effects on the b-wave amplitude in mammalian retina (Table 3). Some authors fail to obtain any effect of the GABA_A_ receptor blockade on the b-wave amplitude (*mouse*: [153];* rabbit*: [229]), while other authors observed its diminution (*cat*: [222, 234]). Still other authors reported that the b-wave amplitude is enhanced during the GABA_A_ receptor blockade (*rat*: [223, 225];* rabbit*: [231];* cat*: [235]; bovine: [236]). Dong and Hare [232] argue that inhibition mediated by GABA_A_ receptors contributes significantly to the kinetics of the scotopic b-wave, but not to its amplitude. They have shown that application of selective GABA_A_ antagonists accelerates the kinetics of the scotopic b-wave without altering its amplitude in rabbit retina. The inconsistency of the results presented probably does not originate in different conditions of light adaptation, because the blockade of GABA_A_ receptors under scotopic conditions of illumination has no effect on the ERG b-wave in mouse retina [153] but enhances it in rat retina [223]. The same is true for cat retina, where the GABA_A_ receptor blockade has opposite effects on the b-wave amplitude in conditions of dark adaptation, decreasing it [222] or increasing it [235]. The action of bicuculline on the scotopic b-wave amplitude appears to be independent of stimulus intensity. It has been demonstrated that its depressing action in cat retina [222] as well as its enhancing action in rat retina [223] is evident over the entire intensity range studied. Species specific differences is unlikely to account for the reported conflicting results, because they have been obtained in similar species (mouse and rat) or even in one and the same species (cat). There are no available data concerning the effects of GABA_A_ antagonists on the mammalian d-wave. More studies are needed to elucidate the role of endogenous GABA, acting through GABA_A_ receptors in modulating the ERG ON and OFF responses in mammalian retina.

The action of GABA mediated by GABA_A_ receptors upon the ERG ON and OFF responses is better established in nonmammalian retina. Wachtmeister [255] reported that bicuculline does not change the b-wave amplitude over the entire intensity range studied in dark adapted mudpuppy retina. Most of the other authors, however, have seen an enhancement of the b-wave amplitude during the GABA_A_ receptor blockade under different conditions of light adaptation (*frog*: [111, 240–242];* turtle*: [111, 250];* fish*: [256]). The effects on the d-wave amplitude are similar to those on the b-wave but expressed to a different degree [111, 240, 242, 250, 256]. We have shown that the enhancing effect of bicuculline is more pronounced on the OFF than ON response over the entire intensity range in conditions of dark adaptation [250]. The same ON-OFF asymmetry of its action has been seen in the lower intensity range under conditions of light adaptation, while the reverse ON-OFF asymmetry has been demonstrated at higher intensities in light adapted eyes. Thus, it appears that GABA_A_ receptors mediate inhibitory influences upon the ON and OFF channels in distal frog retina, whose relative strength depends on the stimulus intensity. However, Bonaventure et al. [241] argue that the GABA_A_ receptor blockade has qualitatively different effect on the ON and OFF responses in frog ERG. While bicuculline and SR95103 (GABA_A_ antagonist) enhance the b-wave amplitude, they have no effect (bicuculline) or markedly diminish (SR95103) the d-wave amplitude.

#### 5.3.2. Effects of GABA_C_ Receptor Antagonists

In mammalian retina some authors fail to obtain any significant effect of GABA_C_ receptor blockade on the scotopic b-wave amplitude (*rabbit*: [232]). This is consistent with the unaltered b-wave amplitude in dark adapted knockout mice lacking GABA_C_ receptors [150, 159]. Although the b-wave amplitude is not significantly changed, the time characteristics of the response are altered, when GABA_C_-mediated transmission is compromised. McCall et al. [159] have observed that the b-wave implicit time is shortened in GABA_C_R knockout mice, while Dong and Hare [232] reported its lengthening under the influence of TPMPA, especially at lower stimulus intensities. While the precise reasons for the discrepancy are not known, differences in experimental conditions seem to be a contributing factor. Dong and Hare [232] blocked acutely the GABA_C_ receptor in adult rabbits, while McCall et al. [159] used knockout approach, in which developmental changes and/or remodeling of other inhibitory transmitter systems may occur. Examination of the kinetics of the scotopic b-wave after blocking acutely the GABA_C_ receptor in the adult wild-type mouse may help to determine whether the role of the GABA_C_ feedback in temporal tuning of scotopic responses in the mouse is fundamentally different from that in the rabbit. Dong and Hare [232] argue that one of the most important functions of the GABA_C_ input to rod bipolar cells is to make the scotopic b-waves more transient, especially those elicited by dim stimuli. However, results of many other authors indicate that the GABA_C_ receptor blockade significantly diminishes the amplitude of the scotopic b-wave in addition to slowing down its kinetics (*rodents*: [153, 223, 224, 226, 227];* rabbit*: [233]) (Table 3). The sensitivity and operational range of the scotopic b-wave are also reduced in GABA_C_ receptor knockout mice and in wild type mice with pharmacologically blocked GABA_C_ receptors [153]. The effects are similar on the light adapted b-wave, indicating that the conditions of light adaptation are not of critical importance for the GABA_C_ receptor-mediated action of GABA. Herrmann et al. [153] proposed that the sustained GABA_C_ receptor-mediated input sensitizes the rod-driven vision by making the rod ON bipolar cell responses larger and operating over a broader light intensity range. The role of GABA acting on GABA_C_ receptors is possibly different in bovine retina, where GABA_C_ receptor blockade leads to an enhancement of the b-wave amplitude [236]. There are no available data concerning the effects of isolated GABA_C_ receptor blockade on the d-wave of mammalian ERG.

In a few studies the effects of isolated GABA_C_ receptor blockade on the ERG waves have been investigated in nonmammalian retina. Behrend et al. [256] reported that the GABA_C_ receptor blockade has little effect on fish ERG (Table 3). Our results on frog and turtle retina indicate that GABA_C_ receptors mediate inhibitory influences upon the ERG ON and OFF responses over the whole intensity range studied in conditions of both dark and light adaptation [111, 242, 250]. These inhibitory effects are more pronounced in conditions of dark than light adaptation. In dark adapted frog retina the enhancing effect of GABA_C_ receptor blockade on the b-wave amplitude does not depend on stimulus intensity, while that on the d-wave is most pronounced in the range of low to moderate intensities [242]. In cone-dominated turtle retina the GABA_C_-mediated effects show clear dependence on stimulus intensity and wavelength [250]. They are most pronounced on the amplitude of both b- and d-waves, when the responses are obtained with low intensity short wavelength stimuli. Thus, it appears that one of the functions of GABA_C_-mediated inhibition in turtle retina is to decrease the sensitivity of ON and OFF responses to blue stimuli. The GABA_C_ receptor blockade widens the dynamic range of the ON response to short (450 nm, 520 nm, and 550 nm), but not long (568 nm, 620 nm, and 650 nm) wavelength stimuli. This indicates that GABA_C_ receptors are involved in keeping relatively high steepness and thus contrast sensitivity to stimuli in the blue end of the spectrum. We have shown that GABA_C_ receptor blockade causes a significant delay of the implicit time and the decay phase of both b-and d-waves, indicating that GABA_C_ receptors are involved in speeding the time course of these responses. This is consistent with the idea that GABA_C_-mediated inhibition limits the bipolar cell output to third-order neurons.

#### 5.3.3. Effects of Simultaneous GABA_A_ and GABA_C_ Receptor Blockade

The role played by GABA, acting on the both types of ionotropic GABA receptors, in shaping the ERG responses is not yet resolved. In mammalian retina some authors reported that picrotoxin (GABA_A_ and GABA_C_ receptor antagonist) decreases the amplitude and delays the descendant part of the scotopic b-wave (*rabbit*: [229, 233];* cat*: [221]) (Table 3). The effects do not depend on the stimulus intensity and are evident over the entire intensity range studied [221]. These results suggest that endogenous GABA, acting on both GABA_A_ and GABA_C_ receptors, increases the amplitude and speeds up the time course of the scotopic ON response. An opposite suggestion could be made based on the results of Gottlob et al. [231]. They have demonstrated that the amplitude of the scotopic rabbit b-wave increases over the entire intensity range under the influence of picrotoxin. No explanation can be offered why picrotoxin has opposite effects on the b-wave amplitude in one and the same species under similar conditions of light adaptation. An enhancement of the b-wave amplitude during the combined GABA_A_ and GABA_C_ receptor blockade has also been seen in bovine retina [236]. Interesting results have been presented by Kapousta-Bruneau [223] in rat retina, where picrotoxin has no effect on the b-wave amplitude, because the selective GABA_A_ and GABA_C_ receptor antagonists have opposite effects and thus their action cancel out each other. These results suggest that the opposite changes of the b-wave amplitude, obtained during simultaneous blockade of GABA_A_ and GABA_C_ receptors, could be due to different proportions of GABA_A_ and GABA_C_ receptor-mediated components. However, we could not find any other study, where such an opposite action of GABA_A_ and GABA_C_ receptor antagonists on the b-wave amplitude has been demonstrated. There are no available data concerning the effects of combined GABA_A_ and GABA_C_ receptor blockade on the mammalian d-wave.

**Figure 3:**
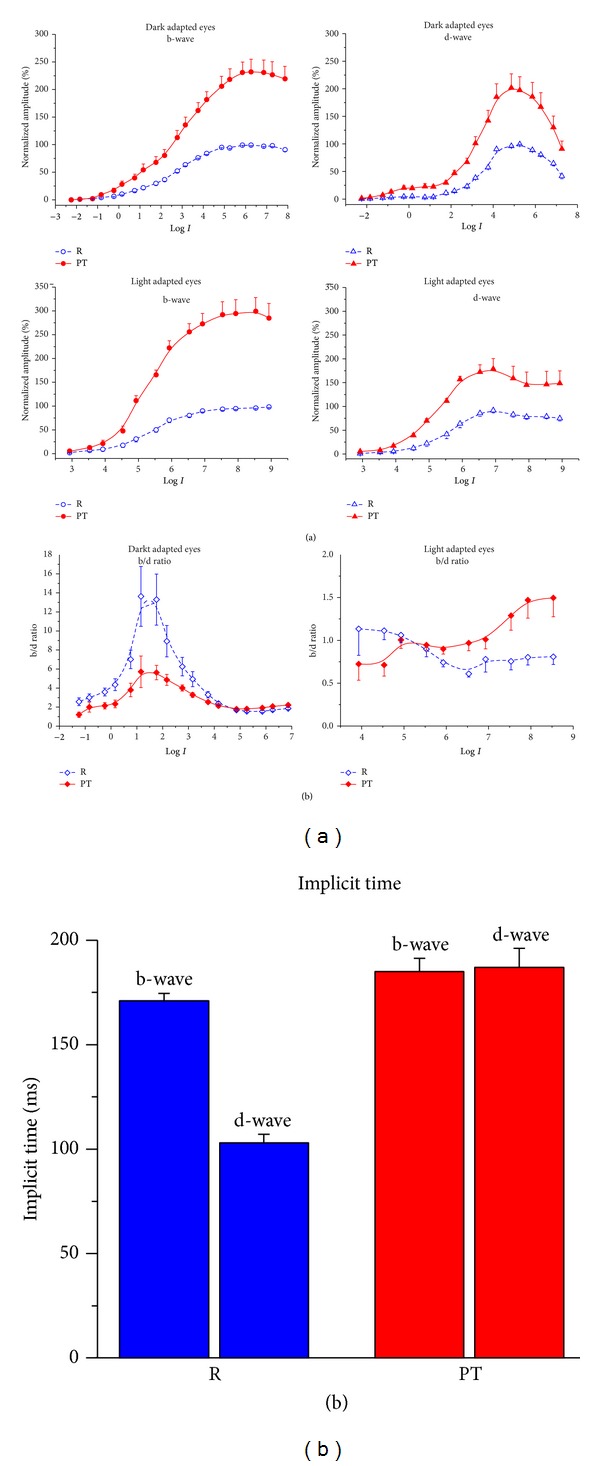
Effects of picrotoxin on the b- and d-wave *V*-log *I* function in dark and light adapted frog eyes. The amplitudes of the b- and d-waves are normalized to *V*_max⁡_ of the responses obtained in the control experiments (R). (a) Changes of the b/d amplitude ratio under the influence of picrotoxin are also shown. (b) Changes of the b- and d-wave implicit time under the influence of picrotoxin in light adapted eyes. Mean values ± SEM are shown.

In nonmammalian retina some authors fail to obtain any effect of picrotoxin on the amplitude of the b-wave (*mudpuppy:* [255]), while other authors reported that picrotoxin reduces the b-wave substantially at all flash intensities (*fish*: [257];* chick*: [238]). Simultaneous intracellular horizontal cell recordings show no effect on their light responses, suggesting that photoreceptor feedback is not involved in the picrotoxin inhibitory effect on the fish ERG [257]. There are much more works, however, where an increase of the b-wave amplitude has been observed under the influence of picrotoxin (*frog*: [239, 241, 243–248];* Xenopus*: [249];* turtle*: [251–254];* fish*: [258, 259]) (Table 3). The effect is evident over the entire range of stimulus intensities in conditions of both dark and light adaptation, indicating that it does not depend on the type of the photoreceptor input (Figure 3). Picrotoxin increases the relative sensitivity of the b-wave (determined by semisaturation constant) in light adapted amphibian eyes [243, 249], but not in the dark [243]. De Vries and Friedman [239], however, argue that this effect of picrotoxin is evident in dark adapted frog eyes as well. Because they have used much narrower intensity range (~5 log units versus ~10 log units in Popova 228), the *V*_max⁡_ value and position of the intensity-response function along the intensity axis may not be accurately determined. It has been shown that picrotoxin significantly delays the latency, implicit time, and decay phase of the b-wave in amphibian and turtle retina [111, 243, 244, 251].

The effects of picrotoxin on the d-wave resemble those on the b-wave (Table 3). Most of the authors reported that picrotoxin enhances the amplitude and delays the time course of the d-wave [215, 243–247, 249, 251–254, 259], although Bonaventure et al. [241] do not find any effect on it (Table 3). Picrotoxin reveals a prominent OFF component in all rod retina of skate [258] and in dark adapted toad retina [248]. Thus, it appears that both the ON and OFF responses in nonmammalian ERG are under tonic inhibitory GABAergic influences. We have shown that their relative strength depends on the stimulus intensity and conditions of light adaptation. This fact is responsible for some of the ON-OFF asymmetries concerning the sensitivity and time course of the ERG responses. We have demonstrated that the GABAergic inhibitory effect is stronger on the amplitude of the OFF in comparison with the ON response in three cases: (a) when the responses are mediated by red rods; (b) when the responses are mediated by cones in the lower intensity range, and (c) in the intensity range, where transition from rod to cone dominated responses occurs in frog retina [243]. However, the GABAergic inhibitory influence is stronger upon the ON than OFF response amplitude, when the responses are (a) mediated by green rods and (b) mediated by cones in the higher intensity range. Thus, it appears that the b/d amplitude ratio, obtained at different stimulus intensities and conditions of adaptation, depends in a critical manner on the retinal GABAergic neurotransmission (Figure 3). The GABAergic neurotransmission is essential also for the ON-OFF asymmetry in the relative sensitivity of the ERG responses. We have found that picrotoxin increases the relative sensitivity of the d-wave in all conditions of adaptation (scotopic, mesopic, and photopic) and thus significantly diminishes (dark adapted eyes) or fully eliminates (light adapted eyes) the difference between the relative sensitivity of the ON and OFF responses. This statement is opposite to that of Arnarsson and Eysteinsson [249], who argue that the small difference in relative sensitivity between ON and OFF response has been further enhanced by the GABAergic blockade in light adapted frog retina. The GABAergic system plays a role in establishing the ON-OFF asymmetry in the time characteristics of the ERG responses. We have shown that the initial difference between the implicit times of the cone mediated b- and d-waves is eliminated under the influence of picrotoxin [243] (Figure 3). Thus, the GABAergic system is responsible for the faster time course of the cone-dominated OFF in comparison with the ON response in frog ERG.

## 6. Conclusion

It is well established that GABA is an inhibitory neurotransmitter released in both the outer and inner plexiform layers of the vertebrate retina. Its actions are mediated in great part by ionotropic GABA_A_ and GABA_C_ receptors, which are well characterized and localized in many retinal neurons. The ionotropic GABA receptors mediate large chloride currents in bipolar cell axon terminals and dendrites, but the exact impact of these currents on light responses of the ON and OFF bipolar cells still needs to be elaborated. The same is true for the involvement of the endogenous GABA in antagonistic centre-surround organization of bipolar cell receptive fields especially in mammalian retina. It has been demonstrated that the GABAergic system plays a role in establishing the ON-OFF asymmetry concerning the time course and absolute and relative sensitivity of the ERG responses obtained under different conditions of light adaptation in amphibian retina. It remains to be determined if endogenous GABA acting through ionotropic GABA receptors plays such a role in mammalian retina including human one. Are the asymmetries in the amplitude of the responses arising from different number of their generators or are they due to different strength of the inhibitory GABAergic influences as it has been shown for the amphibian retina? How the different types of GABA receptors are involved in the spectral coding of visual signals in distal mammalian retina? Further studies are needed to answer these questions.

## Figures and Tables

**Table 1:** GABA receptor expression in retinal neurons, autoradiography, immunocytochemistry, and *in situ* hybridization.

Retinal cell type	GABA_A_R	Reference	Species	GABA_c_R	Reference
Photoreceptors	+	[106, 109, 110]	Fish		
			Frog	+	[111]
	+	[112]	Tiger salamander		
			Rat	+	[113]
			Mouse	+	[113]
	+	[114]	Cat	+	[81]
	+	[115]	Rabbit		
	+	[116]	Monkey		
	+	[116]	Human		

Bipolar cells	+	[111]	Frog	+	[111]
	+	[111]	Turtle	+	[111]
	+	[110]	Fish	+	[110, 117, 118]
			Chick	+	[117, 118]
	+	[119]	Rabbit	+	[117, 120, 121]
	+	[114]	Cat	+	[117, 118, 120]
	+	[121–124]	Rat	+	[117, 120, 124]
	+	[125]	Mouse	+	[125]
	+	[116, 126, 127]	Monkey	+	[117, 120, 127]
	+	[116]	Human		

Horizontal cells	+	[112]	Tiger salamander		
	+	[110]	Fish	+	[110]
	+	[122]	Rat		

Amacrine cells			Frog	+	[111]
	+	[109]	Fish		
	+	[117, 123, 128]	Rat		
	+	[119]	Rabbit		
	+	[129, 130]	Cat		

Ganglion cells	+	[117, 122–124, 131]	Rat		
	+	[119, 131, 132]	Rabbit	+	[132]
	+	[129]	Cat		
	+	[126, 133]	Monkey		

**Table 2:** Effects of GABA and GABA antagonists on the light responses and centre-surround organization of the receptive fields of the ON and OFF bipolar cells.

BCs type	Effects on light responses			Effects on centre-surround organization	Species	References
	GABA	GABA_A_R antagonists	GABA_C_R antagonists			
ON BCs	Decreased	Increased	Increased	Preserved	Mudpuppy	[177]
		Decreased		Eliminated	Mudpuppy	[178]
	Slight reduction			Eliminated	Xenopus	[179]
	No effect				Carp	[180]
		Increased∗			Rat	[181]

OFF BCs	Decreased	No effect	No effect	Preserved	Mudpuppy	[177]
		No effect			Mudpuppy	[178]
	Decreased			Preserved	Xenopus	[179]
	Decreased				Carp	[180]
	Decreased	Decreased	Decreased	Preserved	Tiger salamander	[182]
	Decreased			Eliminated	Tiger salamander	[183]

*Combined application of GABA_A_ + GABA_C_ antagonists.

**Table 3:** Changes of the amplitude of the ERG b- and d-waves under the influence of GABA and antagonists of ionotropic GABA receptors.

ERG wave	GABA	GABA_A_R antagonists	GABA_C_R antagonists	GABA_A_R + GABA_C_R antagonists	Species	Reference
b-wave	No effect				Rat	[219]
	Decrease		decrease		Rat	[224]
		Increase			Rat	[225]
	Increase	Increase	Decrease	No effect	Rat	[223]
			Decrease		Rat, mouse	[226, 227]
	Increase				Mouse	[228]
	Increase	No effect	Decrease		Mouse	[153]
	Increase	No effect		Decrease	Rabbit	[229]
	Decrease				Rabbit	[230]
	Decrease	Increase		Increase	Rabbit	[231]
		No effect	No effect		Rabbit	[232]
			Decrease	Decrease	Rabbit	[233]
	Increase			Decrease	Cat	[221]
	Increase	Decrease			Cat	[222]
		Decrease			Cat	[234]
		Increase			Cat	[235]
		Increase	Increase	Increase	Cow	[236]
	Decrease				Chick	[237]
				Decrease	Chick	[238]
	Decrease			Increase	Frog	[239]
	Decrease	Increase			Frog	[240]
	Decrease	Increase	Increase		Frog	[111]
		Increase		Increase	Frog	[241]
		Increase	Increase		Frog	[242]
				Increase	Frog	[243–248]
	Decrease			Increase	Xenopus	[249]
	Decrease	Increase	Increase		Turtle	[111]
		Increase	Increase		Turtle	[250]
				Increase	Turtle	[251–254]
		No effect	No effect	No effect	Mudpuppy	[255]
		Increase	No effect		Fish	[256]
		No effect		Decrease	Fish	[257]
				Increase	Fish	[258, 259]

d-wave	Decrease	Increase			Frog	[240]
	Decrease	Increase	Increase		Frog	[111]
		Increase	Increase		Frog	[242]
		No effect or decrease		No effect	Frog	[241]
				Increase	Frog	[215, 243–247]
	Decrease			Increase	Xenopus	[249]
	Decrease	Increase	Increase		Turtle	[111]
		Increase	Increase		Turtle	[250]
				Increase	Turtle	[251–254]
		Increase	No effect		Fish	[256]
				Increase	Fish	[259]

## Abbreviations

ERG: Electroretinogram
GABA: Gamma-aminobutyric acid
GAD: Glutamate decarboxylase
OPL: Outer plexiform layer
IPL: Inner plexiform layer
BC: Bipolar cell
GABA_A_R: GABA_A_ receptors
GABA_C_R: GABA_C_ receptors
TPMPA: (1,2,5,6-Tetrahydropyridin-4-yl)methylphosphinic acid.
